# Supplementary material for: Urease of Aspergillus fumigatus Is Required for Survival in Macrophages and Virulence
Source: Microbiol Spectr. 2023 Mar 14;11(2):e03508-22. doi: 10.1128/spectrum.03508-22 (PMC10100864; doi:10.1128/spectrum.03508-22)
Supplement: Supplemental file 1 — Supplemental material. Download spectrum.03508-22-s0001.pdf, PDF file, 14.7 MB [file spectrum.03508-22-s0001.pdf]

Table S1: Plasmids used in this study

Plasmid	Description*	Source
pUChph	Amp^R; Plasmid containing hph cassette conferring resistance to hygromycin	(1)
pSK275	Amp^R; Plasmid containing ptrA cassette conferring resistance to pyrithiamine	(2)
pME2967	Amp^R; Plasmid containing cTap tag	(3)
pME2968	Amp^R; Plasmid containing nTap tag	(3)
pFastBac_EGFP	Amp^R; Plasmid carrying EGFP-encoding sequence	Zhang Y, Zhejiang Univ.
pnEA_vStrep	Amp^R; *E. coli* overexpression vector	(4)
ppCS	Sm^R; *E. coli* overexpression vector	(4)
pMalC2HTEV	Amp^R; Plasmid carrying the maltose-binding protein (MBP) gene	Unpublished
pME2968_ nTap-ureB	Amp^R; Derivative of pME2968, with ureB genomic DNA at the 3' end of nTap	This study
pME2968_ nTap-ureF	Amp^R; Derivative of pME2968, with ureF genomic DNA at the 3' end of nTap	This study
pME2967_ cTap-ptrA	Amp^R; Derivative of pME2967, with ptrA cassette downstream of cTap	This study
pFastBac_ureB5'arm-EGFP	Amp^R; Derivative of pFastBac_EGFP, with 5'UTR of *ureB* at the 5' end of EGFP	This study
pnEA_vStrep-UreB	Amp^R; pnEA_vStrep derivative expressing Strep-UreB	This study
pMalC2HTEV-UreD	Amp^R; Plasmid containing MBP-UreD fusion	This study
ppCS_MalC2HTEV-UreD	Sm^R; ppCS derivative expressing MBP-UreD fusion	This study
ppCS_His-UreF	Sm^R; ppCS derivative expressing His-UreF	This study
ppCS_UreG-HA	Sm^R; ppCS derivative expressing UreG-HA	This study
pnEA_vStrep-UreB/UreG-HA	Amp^R; pnEA_vStrep derivative co-expressing Strep-UreB and UreG-HA	This study
ppCS_MBP-UreD/His-UreF	Sm^R; ppCS derivative co-expressing MBP-UreD and His-UreF	This study
ppCS_MBP/His-UreF	Sm^R; ppCS derivative co-expressing MBP and His-UreF	This study
pnEA_vStrep-UreB/HA	AmpR; pnEA_vStrep derivative expressing Strep-UreB-HA	This study

*Amp, ampicillin; Sm, streptomycin.

Table S2: Primers used in this study.

No.	Primer	Sequence (5'>3')	Purpose
P1	ureB_F	GTTTGGCTTGTGCATCTCCTGG	Δ*ureB* mutant (PtrA^R)
P2	ureB_ptrA_R	GGCCTGAGTGGCCATCGAATTCAGATGACGAGCTTGTCCAGC	
P3	ureB_ptrA_F	GAGGCCATCTAGGCCATCAAGCGATGTCCTCGGATTCGCAGG	
P4	ureB_R	TGGATCGGTATCACTCAAACGC	
P5	ureBN_F	CCTGGTTGATAGTTCTGTTGCC	
P6	ureBN_R	GCTGATGCTAAACAAACAGTGG	
P7	ureB (XbaI)_F	ACGTTCTAGAAATTGGGAACAGCTCTTTGGATTGTTCATAGTTGTCG	Complemented *ureB* strain (Hyg^R)
P8	ureB (XbaI)_R	ACGTTCTAGACTAGTAAACGAAGTAATCCTGCGTC	
P9	ureD_F	ACCCAGCATTGACAAGCGAG	Δ*ureD* mutant (ptrA^R)
P10	ureD_ptrA_R	GGCCTGAGTGGCCATCGAATTCGGGGATTAGGATGTAGGGAG	
P11	ureD_ptrA_F	GAGGCCATCTAGGCCATCAAGCCTCGTGTTGATGGCGACTGC	
P12	ureD_R	CCCATCACCCACTTCCATGC	
P13	ureDN_F	GCCCTATGTCTCTCAGAATGCG	
P14	ureDN_R	ATGGCAGCGTGTGATGGAG	
P15	ureD (HindIII)_F	ACGTAAGCTTGCTGCATAAGCTGACCCAGC	Complemented *ureD* strain (Hyg^R)
P16	ureD (HindIII)_R	ACGTAAGCTTCTCGGTTTTATCGACGGTCG	
P17	ureF_F	CATCGAGGTAGCCAGCCCAC	Δ*ureF* mutant (ptrA^R)
P18	ureF_ptrA_R	GGCCTGAGTGGCCATCGAATTCTGTTCTTCCCATTCATTCGG	
P19	ureF_ptrA_F	GAGGCCATCTAGGCCATCAAGCCACTTGAGCACCTACATGGG	
P20	ureF_R	AAGCGCGAGAGTTGGAGG	
P21	ureFN_F	TCAGTCATCTTGTAGGTATCGC	
P22	ureFN_R	TCGCAACTAAAGGAATCGGAGG	
P23	ureF (XbaI)-F	ACGTTCTAGACGCAGCTTGAGCTGTTAATATTAAGCCC	Complemented *ureF* strain (Hyg^R)
P24	ureF (XbaI)-R	ACGTTCTAGAGTGGTAGACAGTTTCCCATGTAGGTG	
P25	ureG_F	TCTTGTCCTGAGTTGCGAGTGG	Δ*ureG* mutant (ptrA^R)
P26	ureG_ptrA_R	GGCCTGAGTGGCCATCGAATTCCTGTAGTGAGGTGGGATGGC	
P27	ureG_ptrA_F	GAGGCCATCTAGGCCATCAAGCATCCCCATACTGCCATGCAC	
P28	ureG_R	TTGCTTCTTTGCTCATCTCCGC	
P29	ureGN_F	TTCACGTTGAATATGAGGTCCTC	
P30	ureGN_R	TGCTCATCTCCGCGAGAGG	
P31	ureG (XbaI)-F	ACGTTCTAGAGAGATGGTTTCCGTTTTCGC	Complemented *ureG* strain (Hyg^R)
P32	ureG (XbaI)-R	ACGTTCTAGACAAGTCTTGGATATTTTGCTGC	
P33	ptrA-F	GAATTCGATGGCCACTCAGGCC	Pyrithiamine-resistant marker
P34	ptrA-R	GCTTGATGGCCTAGATGGCCTC	
P35	nTap_I	CATATGGGAGAGCTCCCAACGC	nTap-homologous recombination
P36	nTap _II	ACCACCGCTACCACCGTCGACGGTATCGATAAGCTTATCGTC	
P37	nTap _ureB_I	TCGACGGTGGTAGCGGTGGTATGCACCTAATTCCCAAGGAG	pME2968_ nTap-ureB plasmid (Amp^R)
P38	nTap _ureB_II	GTTGGGAGCTCTCCCATATGCTAGTAAACGAAGTAATCCTGCGTCAAGGGC	
P39	nTap _ureF_I	TCGACGGTGGTAGCGGTGGTATGAATGGGAAGAACAGTATACAAGCTTTGG AG	

P40	nTap _ureF_II	TGGGAGCTCTCCCATATGTTATGAATTGAATATTCTGCTATAGAGAAGCTCATGCCGCCC	pME2968_ nTap-ureF plasmid (Amp^{R})
P41	nTap _ureB_1	CGAGAAGAAGAAGCGCACGTC	*nTap*::*ureB* strain (Hyg^{R})
P42	nTap _ureB_2	CGTGTTGCGCAAGGCCTGCCATGGTAACAGGTGCAGGAGCCAG	
P43	nTap _ureB_3	CTGGCTCCTGCACCTGTTACCATGGCAGGCCTTGCGCAACACG	
P44	nTap_ureB_4	GCAACGCCCTTTGGAGAGCTCTAGTAAACGAAGTAATCCTG	
P45	nTap_ureB_5	CAGGATTACTTCGTTTACTAGAGCTCTCCAAAGGGCGTTGC	
P46	nTap_ureB_6	GCAAGCGCACGCACTCGCGTGGAGCCAAGAGCGG	
P47	nTap_ureB_7	TCTTGGCTCCACGCGAGTGCGTGCGCTTGCAACTTGCAC	
P48	nTap_ureB_8	AAGGAGATGTGGTCGGACGA	
P49	nTap_ureBN_1	CGCAAGGGCAATGAAAAGGAC	
P50	nTap_ureBN_8	CCATACTCACGCCATTCCTCC	
P51	nTap_ureF_1	CATCGAGGTAGCCAGCCCAC	*nTap*::*ureF* strain (ptrA^{R})
P52	nTap_ureF_2	CGTGTTGCGCAAGGCCTGCCATTCGGACATTCCTCAGTGC	
P53	nTap_ureF_3	GCACTGAGGAATGTCCGAATGGCAGGCCTTGCGCAACACG	
P54	nTap_ureF_4	GGGATCCCGTAATCAATTGTTATGAATTGAATATTCTGCTATAGAGAAGCTCATGCCGCCC	
P55	nTap_ureF_5	GGGCGGCATGAGCTTCTCTATAGCAGAATATTCAATTCATAACAATTGATTACGGGATCCC	
P56	nTap_ureF_6	CCCATGTAGGTGCTCAAGTGTCTTTCTTGTTACACATAATTATTCTAGAATGCCCCACCG	
P57	nTap_ureF_7	GAAAGACACTTGAGCACCTACATGGG	
P58	nTap_ureF_8	AAGCGCGAGAGTTGGAGG	
P59	nTap_ureFN_1	TCAGTCATCTTGTAGGTATCGC	
P60	nTap_ureFN_8	TCGCAACTAAAGGAATCGGAGG	
P61	cTap_ptrA_I (SpeI)	ACGTACTAGTGAATTCGATGGCCACTCAGG	pME2967_ cTap-ptrA^{R} plasmid (Amp^{R})
P62	cTap_ptrA_II (PstI)	ACGTCTGCAGAAGCTTGATGGCCTAGATGGC	
P63	ureD_cTap_1	ACCCAGCATTGACAAGCGAG	*ureD*::*cTap* strain (ptrA^{R})
P64	ureD_cTap_2	CTTCTCTTACCACCGCTACCACCTAGACACATAAAAGATTCTTCTCCAAACTCTCTGAGAATTGTCCC	
P65	ureD_cTap_3	GTGTCTAGGTGGTAGCGGTGGTAAGAGAAGATGGAAAAAGAATTTCATAGCCGTC	
P66	ureD_cTap_4	CGAGAAGAACCAGAAAGAGACTAGGGTCTTTCTTGTTACACATAATTATTCTAGAATGCCCCACCG	
P67	ureD_cTap_5	GAAAGACCCTAGTCTCTTTCTGGTTCTTCTCG	
P68	ureD_cTap_6	CCCATCACCCACTTCCATGC	
P69	ureD_cTapN_1	GCCCTATGTCTCTCAGAATGCG	
P70	ureD_cTapN_2	ATGGCAGCGTGTGATGGAG	
P71	ureG_cTap_1	GGGAAAAGACAGAAGGGTACTGC	*ureG*::*cTap* strain (ptrA^{R})
P72	ureG_cTap_2	CCATCTTCTCTTACCACCGCTACCACCTTCATCCACACTCCCCGAATTCTTCGG	
P73	ureG_cTap_3	GGATGAAGGTGGTAGCGGTGGTAAGAGAAGATGGAAAAAGAATTTCATAGCCGTCTCAGC	
P74	ureG_cTap_4	CCCGCTCAATCGTAGATTGATGTCTTCTTTCTTGTTACACATAATTATTCTAGAATGCCCCACCG	
P75	ureG_cTap_5	CAAGAAAGAAGACATCAATCTACGATTGAGCGGG	
P76	ureG_cTap_6	ATGTCGGGCATCAGAATGGG	

P77	ureG_cTapN_1	GGTTTCCGTTTTCGCTTGGG	
P78	ureG_cTapN_2	ACTCCCAAACCAACCTCGTC	
P79	EGFP_I	ATGGTGAGCAAGGGCGAGGAGCTGTTCACC	pFastBac_*ureB*5'UTR-EGFP plasmid (Amp^R)
P80	EGFP_II	CCCGGGTGATCAAGTCTTCGTC	
P81	ureB5'_egfp_I	CGAAGACTTGATCACCCGGGCGAGAAGAAGAAGCGCACGTC	
P82	ureB5'_egfp_II	TCCTCGCCCTTGCTCACCATGGTAACAGGTGCAGGAGCC	
P83	egfp_ureB_1	CGAGAAGAAGAAGCGCACGTC	*EGFP*::*ureB* strain (Hyg^R)
P84	egfp _ureB_2	ACCACCGCTACCACCCTTGTACAGCTCGTCCATGCCG	
P85	egfp_ureB_3	CAAGGGTGGTAGCGGTGGTATGCACCTAATTCCCAAGGAG	
P86	egfp_ureB_4	GCAACGCCCTTTGGAGAGCTCTAGTAAACGAAGTAATCCTG	
P87	egfp_ureB_5	CAGGATTACTTCGTTTACTAGAGCTCTCCAAAGGGCGTTGC	
P88	egfp_ureB_6	GCAAGCGCACGCACTCGCGTGGAGCCAAGAGCGG	
P89	egfp_ureB_7	TCTTGGCTCCACGCGAGTGCGTGCGCTTGCAACTTGCAC	
P90	egfp_ureB_8	AAGGAGATGTGGTCGGACGA	
P91	egfp_ureBN_1	CGCAAGGGCAATGAAAAGGAC	
P92	egfp_ureBN_8	CCATACTCACGCCATTCCTCC	
P93	β-Actin-F	CCACGTCACCACTTTCAACTCC	Real-time PCR
P94	β-Actin-R	CCTTCTGCATACGGTCGGAG	
P95	ureB_qRT_F	TCATCTGGCACCCTAGCACC	
P96	ureB_qRT_R	TACTTGGGACGAAGGCGC	
P97	$UreB_{(NdeI)}$_Fo	ACGTCATATGCACCTAATTCCCAAGGAGCTGGACAAGC	pnEA_vStrep-UreB plasmid
P98	$UreB_{(XbaI)}$_Re	ACGTTCTAGATTAGTAAACGAAGTAATCCTGCGTCAAGGGCA	
P99	$UreD_{(NcoI)}$_Fo	ACGTCCATGGgaATGCCAATAATATCACCCTTTGAGTCC	pMalC2HTEV-UreD plasmid
P100	$UreD_{(HindIII)}$_Re	ACGTAAGCTTttaTAGACACATAAAAGATTCTTCTCC	
P101	$MBP_{(NdeI)}$_Fo	ACGTCATATGAAAATCGAAGAAGGTAAACTGG	ppCS_MalC2HTEV-UreD plasmid
P102	$UreD_{(NheI)}$_Re	ACGTGCTAGCttaTAGACACATAAAAGATTCTTCTCC	
P103	$UreF_{(BamHI)}$_Fo	ACGTGGATCCCATCACCATCATCACCACGGCAGCatgaatgggaagaacagtatacaagctttggag	ppCS_His-UreF plasmid
P104	$UreF_{(XbaI)}$_Re	ACGTTCTAGAttatgaattgaatattctgctatagagaagctcatgcc	
P105	UreGHA _1	ATGTCACACTCCCACTCTCATGACCAC	ppCS_UreG-HA plasmid
P106	UreGHA _2	ttaAGCGTAATCTGGAACATCGTATGGGTATTCATCCACACTCCCCGA	
P107	UreGHA _3	ATGTTCCAGATTACGCTtaaTCTAGAGCTAGCCCTAGG	
P108	UreGHA _4	TCATGAGAGTGGGAGTGTGACATATGTATATCTCCTTC	
P109	pUreG_HA_F	AGGATTACTTCGTTTACtaaTCCTGCATTAGGAGATCTACTAGTCG	pnEA_vStrep-UreB/UreG-HA plasmid
P110	pUreG_HA_R	CTCCTAGGGCTAGCTCTAGAttaAGCGTAATCTGGAACATCGTATGGG	
P111	pUreB_vect_F	TCTAGAGCTAGCCCTAGGAGATCC	
P112	pUreB_vect_R	ttaGTAAACGAAGTAATCCTGCGTC	
P113	mbp/hisUreF_F	ACGTGGTACCCGCGAATGACTTTGAGACGG	ppCS_MBP/His-UreF plasmid
P114	mbp/hisUreF_R	ACGTGGTACCGCTCGAATTAGTCTGCGCGTC	
P115	StrepUreB/ha_1	CATACGATGTTCCAGATTACGCTGGCGAAAACCTTTACTTCCAGGG	pnEA_vStrep-UreB/HA plasmid
P116	StrepUreB/ha_2	GCTGTAGGCATAGGCTTGGT	
P117	StrepUreB/ha_3	ACCAAGCCTATGCCTACAGC	
P118	StrepUreB/ha_4	GTAATCTGGAACATCGTATGGGTAGCTGCTTTTTTCGAACTGCG	
P119	ureB_pro_for	TGGAGGAGCAGTTGTAGAGG	Southern blot

P120	ureB_pro_rev	TACTGTGGATGTGGATGTGG	
P121	ureD_pro_for	ACCAATCCTTACCATACCGC	
P122	ureD_pro_rev	GGGGATTAGGATGTAGGGAG	
P123	ureF_pro_for	TTGCCGCAGCGTAAGGTG	
P124	ureF_pro_rev	ATTCGGACATTCCTCAGTGC	
P125	ureG_pro_for	GTGAGGTGCTTACTTGCTCC	
P126	ureG_pro_rev	CAATGTTAGACAAGGGCGAG	

Table S3: List of accession numbers

Species	Gene	Predicted function	UniProt Accession No.
Aspergillus fumigatus Af293	*AFUA_1G04560*	Urease	Q6A3P9
	AFUA_2G16070	Urease accessory protein UreD	Q4WZR1
	AFUA_6G04385	Uncharacterized	A0A0J5SE93
	AFUA_2G12900	Urease accessory protein UreG, putative	Q4X0N2
Cryptococcus neoformans H99	*CNAG_05540*	Urease	O13465
	CNAG_01166	Urease accessory protein	J9VQV8
	CNAG_02057	Uncharacterized protein	J9VMQ5
	CNAG_00678	Urease accessory protein UreG	J9VEN6
Coccidioides posadasii C735	*CPC735_069440*	Urease	C5P0Q4
	CPC735_058660	Urease accessory protein UreD containing protein	C5PIZ3
	CPC735_036810	Uncharacterized protein	C5P272
	CPC735_064560	Urease accessory protein ureG, putative	C5PBM9
Magnaporthe oryzae strain 70-15	*MGG_01324*	Urease	G4MYL6
	MGG_09264	Urease accessory protein UreD	G4MQ94
	MGG_14268	Urease accessory protein UreF	G4ML33
	MGG_01086	cobW domain-containing protein	G4NCH0
Neurospora crassa ATCC 24698	*NCU03127*	Urease	V5IQN1
	NCU01246	Urease accessory protein UreD	Q1K6K4
	NCU01185	Urease accessory protein UreF	Q1K851
	NCU01511	Urease accessory protein ureG	Q1K5K4
Oryza sativa subsp. japonica	*Os12g0234800*	Urease	B9GCH9
	Os02g0173000	Urease accessory protein D	Q0E3I9
	Os02g0168000	Urease accessory protein F	Q0E3L5
	Os05g0565200	Urease accessory protein G	Q6AUF3
Schizosaccharomyces pombe strain 972	*SPAC1952.11c*	Urease	O00084
	SPAC3A12.09c	Uncharacterized urease accessory protein ureD-like	P87125
	SPAC29A4.13	Uncharacterized urease accessory protein UreF-like	O14016
	SPCPB16A4.05c	Uncharacterized urease accessory protein ureG-like	Q96WV0
Histoplasma capsulatum G186AR	*HCBG_00793*	Urea amidohydrolase	C0NCE7
	HCBG_04490	Urease accessory protein UreD	C0NLW0
	HCBG_08677	Urease accessory protein UreF	C0NZU7
	HCBG_00970	CobW/P47K family protein	C0NCX4
Arabidopsis thaliana	*At1g67550*	Urease	Q9SR52
	At2g35035	Urease accessory protein D	Q7Y0S0
	At1g21840	Urease accessory protein F	Q9XHZ3

	At2g34470	Urease accessory protein G	O64700
Solanum tuberosum	*ure*	Urease	Q93WI8
	ureD	Urease accessory protein D	M1B782
	ureF	Urease accessory protein F	M1C8W7
	ureG	Urease accessory protein G	Q9AR61
Helicobacter pylori ATCC 700392	*HP_0072*	Urease subunit beta	P69996
	HP_0067	Urease accessory protein UreH	Q09067
	HP_0070	Urease accessory protein UreE	Q09064
	HP_0069	Urease accessory protein UreF	Q09065
	HP_0068	Urease accessory protein UreG	Q09066
Haemophilus influenzae ATCC 51907	*HI_0539*	Urease subunit alpha	P44391
	HI_0535	Urease accessory protein UreD	P44397
	HI_0538	Urease accessory protein UreE	P44394
	HI_0537	Urease accessory protein UreF	P44395
	HI_0536	Urease accessory protein UreG	P44396
Proteus mirabilis HI4320	*PMI3685*	Urease subunit alpha	P17086
	PMI3682	Urease accessory protein UreD	P17089
	PMI3686	Urease accessory protein UreE	P17090
	PMI3687	Urease accessory protein UreF	P17091
	PMI3688	Urease accessory protein UreG	Q06206
Staphylococcus aureus NCTC 8325	*SAOUHSC_02561*	Urease subunit alpha	Q2G2K5
	SAOUHSC_02565	Urease accessory protein UreD	Q2G272
	SAOUHSC_02562	Urease accessory protein UreE	Q2G2K8
	SAOUHSC_02563	Urease accessory protein UreF	Q2G2K7
	SAOUHSC_02564	Urease accessory protein UreG	Q2G273
Klebsiella aerogenes	*ureC*	Urease subunit alpha	P18314
	ureD	Urease accessory protein UreD	Q09063
	ureE	Urease accessory protein UreE	P18317
	ureF	Urease accessory protein UreF	P18318
	ureG	Urease accessory protein UreG	P18319

```
A. thaliana  --------------MEEDERRDIVMSRASSCMQWSQWQL-----------------LDSI
A. fumigatus MNGKNSIQALEKEIADLEYRLRNARTRLTLARQEDTRELPSNDEPYLPPNHALLLLSDSA
                            : : *   . :* : . * .  :*                  ** 

A. thaliana  LPTGGFAHSFGLEAAIQT-----RLVSSPEDLETHIIHVLDNTASLLLPFVYSALKSPDI
A. fumigatus LPLGSFAYSSGLESYLAHSKPLPRSVTTIASFHHFLKLSIASIASTSLPYVLAAYRNP--
             ** *.**:* ***: :       * *::  .:. .:   : . **  **:* :* :.*  

A. thaliana  ETWHKLDGILNATLTNQVSSKASMSQGSALFRIAASVFTEVPN-------------LKMI
A. fumigatus GELETLDNDLDASTPCIVAQRASVAQGRALLGVWERAFRSAYASGPPAGETDAAKAVQMI
                ..**. *:*: .  *:.:**::** **: :   .* ..               ::**

A. thaliana  RDASLGSK---------NVCFHHAPIFGLVCGLLGMDSETSQRAYLFVTLRDVLSAATRL
A. fumigatus ENFSDALKSWVGTADELGPKGHLAPLWGVVCLAMGVDLRQTAYVFMLNHAKAVLSAAVRA
             .: * . *         .   * **::*:**  :*:* . :  .:::   : *****.* 

A. thaliana  NIVGPMGAS---VMQHRIAIVTETVLEKWMNREAGEACQTSPLLDVVQGCHGYLFSRLFC
A. fumigatus SVMGPYQAQSVLASKALQAMISERIDREWN-TAVEDAGQIVPPLDLWVGRHELLYSRIFN
             .::**  *.   . :   *:::* : .:*    . :* *  * **:  * *  *:**:* 

A. thaliana  S
A. fumigatus S
             *
```

Fig S1. Alignment of the deduced amino acid sequence of UreF from *A. fumigatus* with the known UreF sequence from *A. thaliana*.

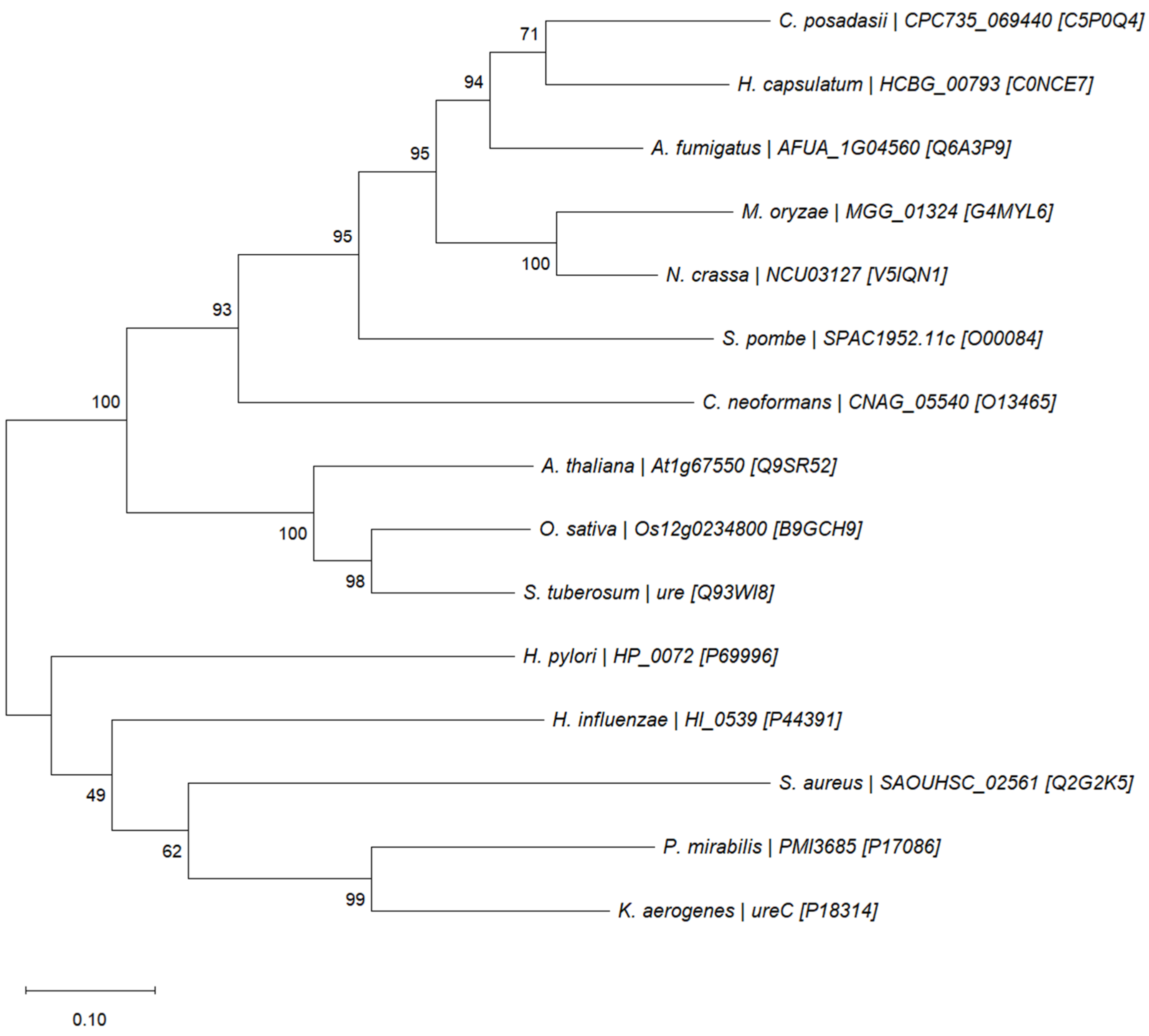

Fig S2. Molecular phylogenetic analysis results for Urease, based on the maximum likelihood method. The evolutionary history was inferred by using the Maximum Likelihood method and Le_Gascuel_2008 model (5). Initial tree(s) for the heuristic search were obtained by applying Neighbor-Join and BioNJ algorithms to a matrix of pairwise distances estimated using the JTT mode. The tree is drawn to scale, with branch lengths measured in the number of substitutions per site. The scale bar represents the number of amino acid substitutions per site; numbers on the dendrogram are percentages of occurrence in 1,000 bootstrapped trees. Evolutionary analyses were conducted in MEGA11(6).

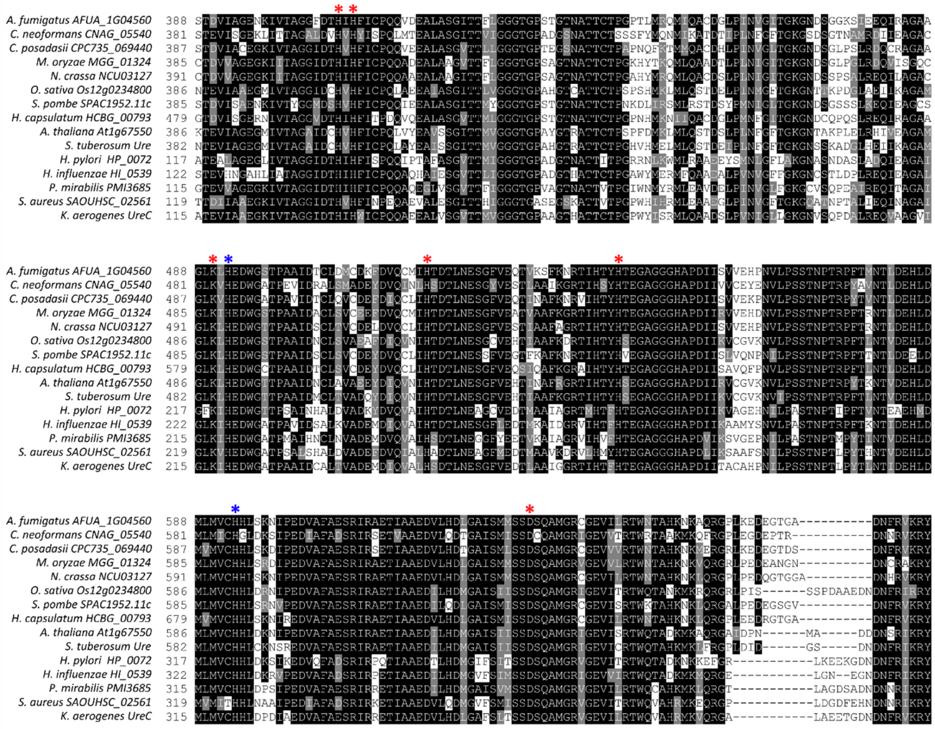

Fig S3. Multiple sequence alignment of the whole urease from fungi and plants with the α subunit of urease from bacteria. Red asterisks indicate the conserved residues (one lysine, four histidine and one aspartate) that form the active site of urease. The urease activation process requires the carbamylation of the active-site lysine followed by insertion of nickel ions. Blue asterisks indicate two extra histidine residues thought to function in substrate binding and/or catalysis. The amino acid sequences were aligned by Clustal W and shaded by BOXSHADE (BoxShade Server (vital-it.ch). All the sequences are from UniProt and the accession numbers are listed in Table S3.

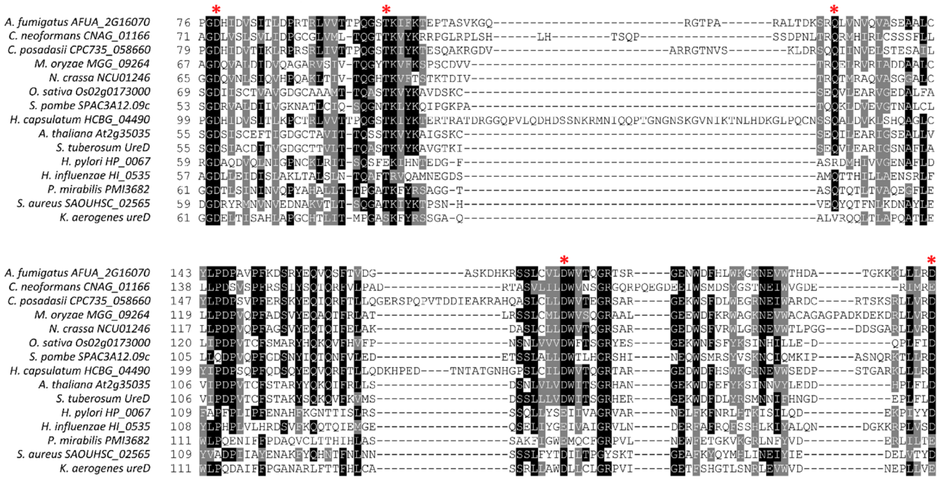

Fig S4. Sequence alignment of the characteristic regions of *A. fumigatus* UreD and its homologs. Asterisks indicate conserved residues that support an internal nickel-transfer tunnel so far identified in bacteria. Accession numbers for the sequences refer to Table S3.

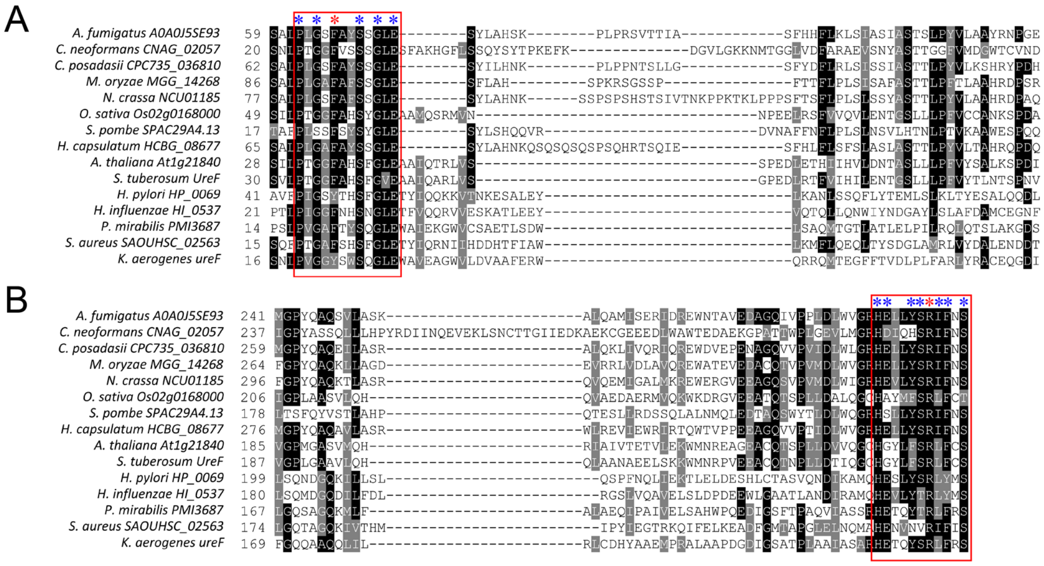

Fig S5. Sequence alignment of the N-terminal (A) and C-terminal (B) regions of *A. fumigatus* UreF and its homologs. The conserved terminal residues thought to make up the UreG-binding site are boxed, of which the most critical ones are marked with red asterisks. Accession numbers for the sequences refer to Table S3.

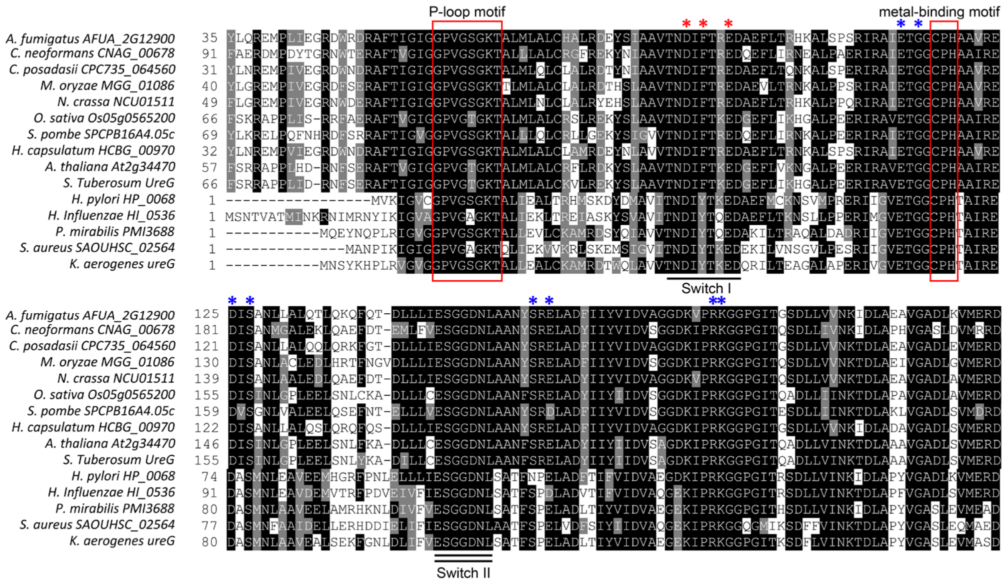

Fig S6. Sequence alignment of the characteristic regions of *A. fumigatus* UreG and its homologs. The P-loop and CPH metal-binding motifs are indicated by solid boxes, two switch regions are underlined and noted. Residues essential for GTP-induced conformational changes and Ni^{2+} binding are indicated by red asterisks, and those likely involved in interaction with UreF are indicated by blue asterisks. The highly conserved functional HXH motif between fungi and plants at the histidine-rich N-terminal tail is indicated by a dashed box. Accession numbers for the sequences refer to Table S3.

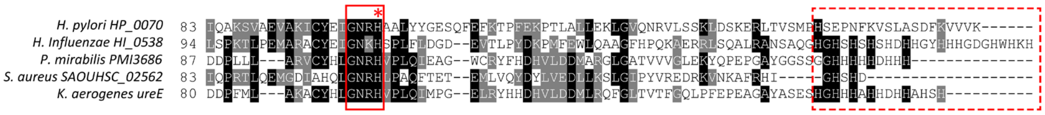

Fig S7. Sequence alignment of the C-terminal regions of UreE from five pathogenic bacteria. The GNXH motif containing a conserved metal-binding his residue (*) is marked by a solid box, the histidine-rich C-terminal tail is marked by a dashed box. Accession numbers for the sequences refer to Table S3.

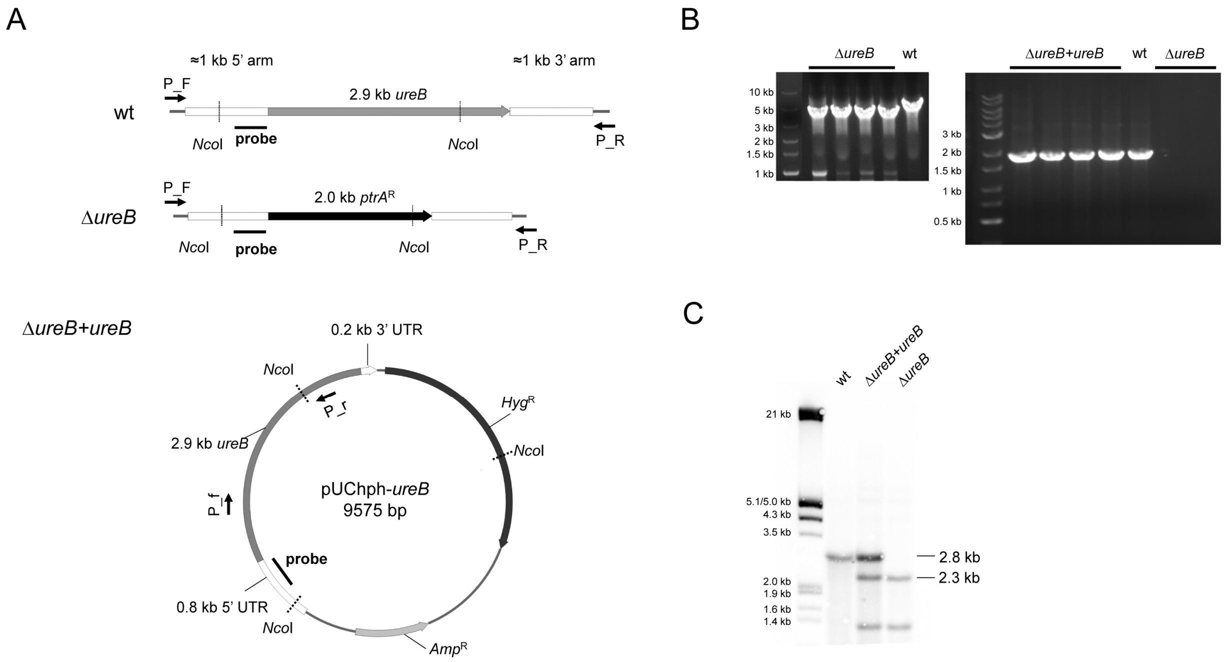

Fig S8. Construction of the mutant and complemented strains of *ureB*. (A) Genome organization of the wild-type, Δ*ureB*, and Δ*ureB*+*ureB* strains. (B&C) Diagnostic PCR and Southern blot analyses of the strains. The Δ*ureB* strain was complemented with the native *ureB* gene by ectopic integration. The primer pairs (P_F/P_R and P_f/P_r) were used for PCR verification of the mutant and complemented strains respectively.

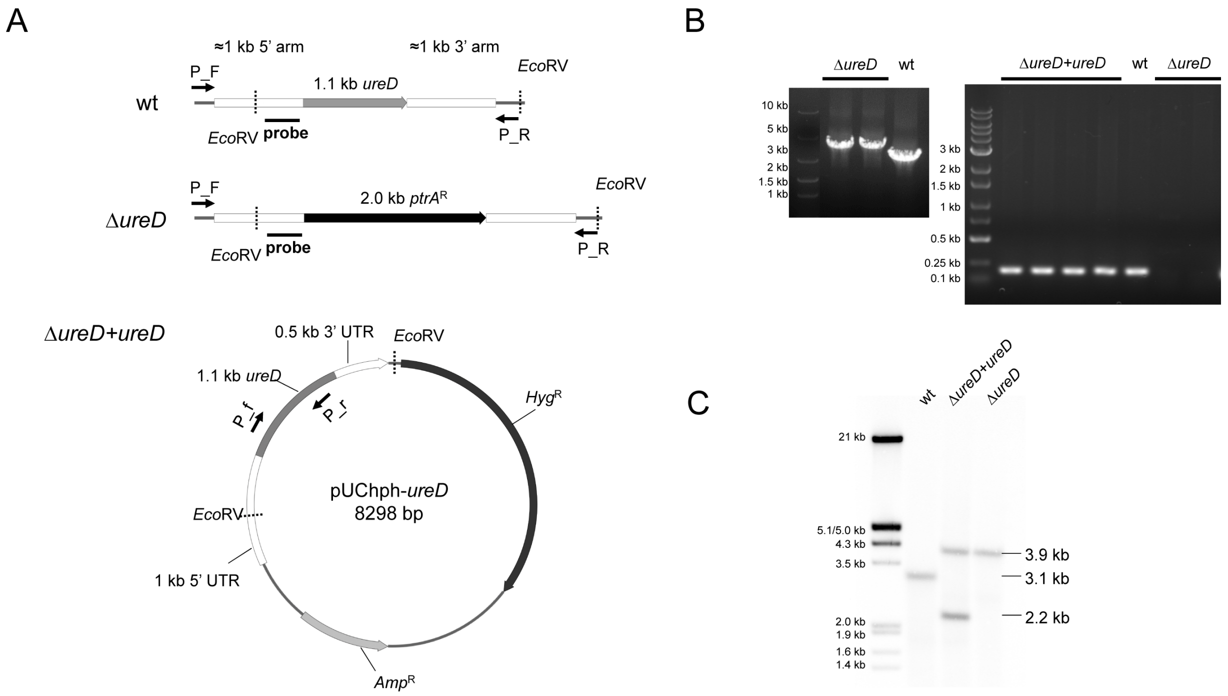

Fig S9. Construction of the mutant and complemented strains of *ureD*. (A) Genome organization of the wild-type, Δ*ureD*, and Δ*ureD*+*ureD* strains. (B&C) Diagnostic PCR and Southern blot analyses of the strains. The Δ*ureD* strain was complemented with the native *ureD* gene by ectopic integration.

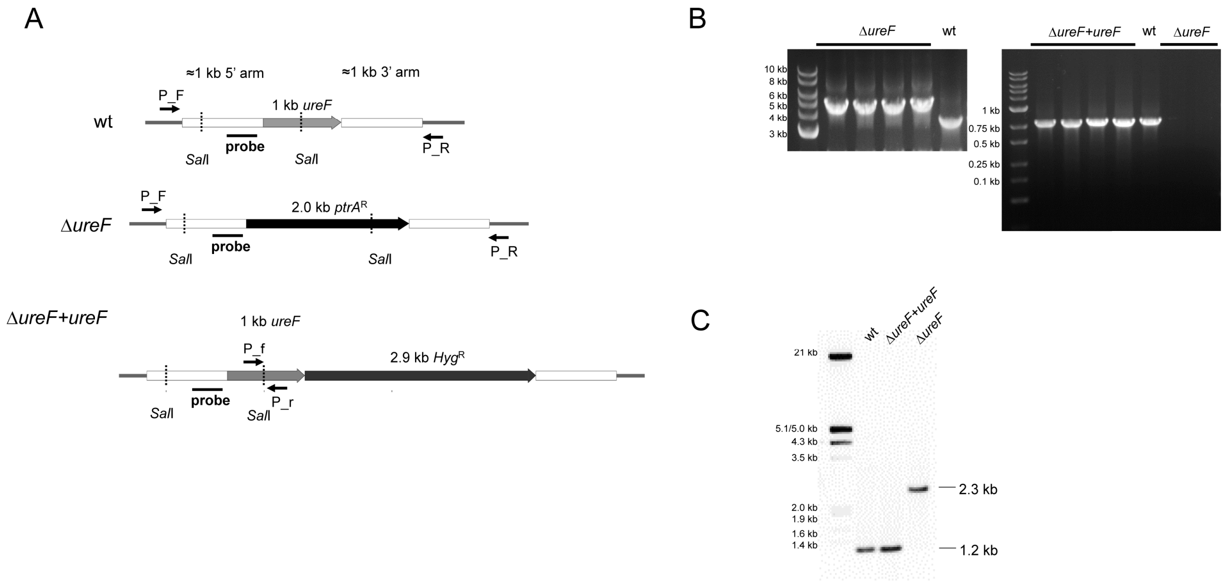

Fig S10. Construction of the mutant and complemented strains of *ureF*. (A) Genome organization of the wild-type, Δ*ureF*, and Δ*ureF*+*ureF* strains. (B&C) Diagnostic PCR and Southern blot analyses of the strains. The Δ*ureF* strain was complemented by homologous recombination with the native *ureF* gene fused with a Hyg^R cassette.

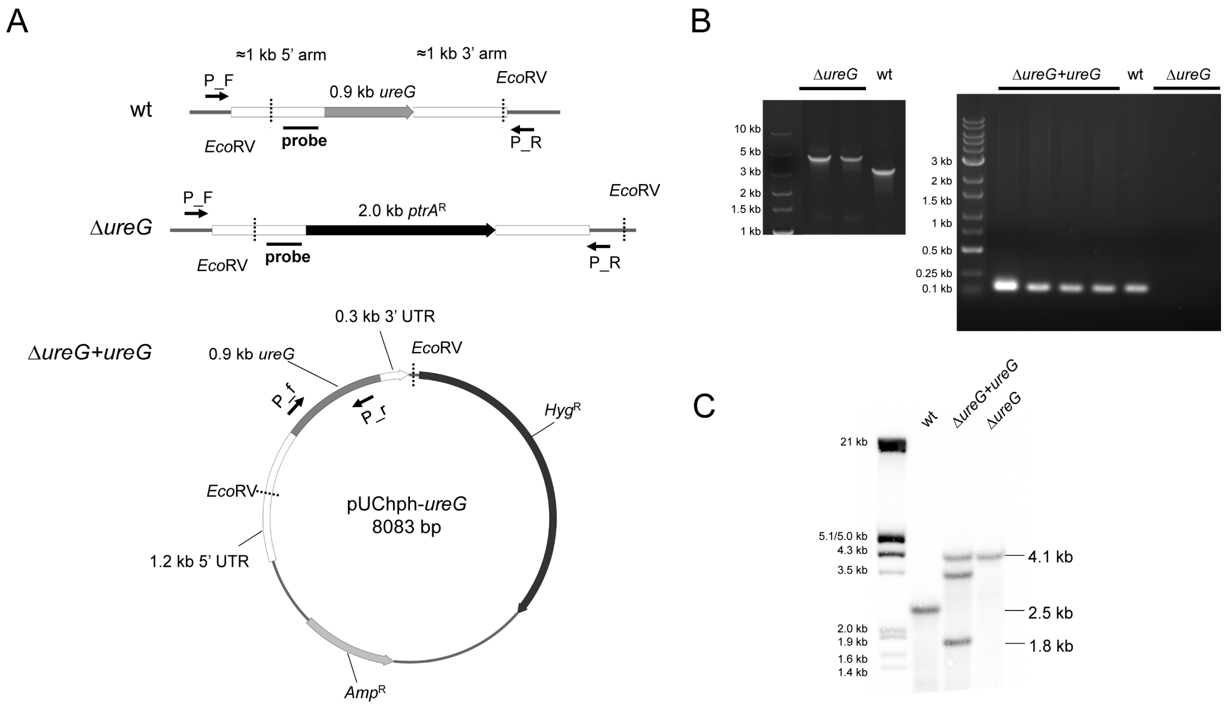

Fig S11. Construction of the mutant and complemented strains of *ureG*. (A) Genome organization of the wild-type, Δ*ureG*, and Δ*ureG*+*ureG* strains. (B&C) Diagnostic PCR and Southern blot analyses of the strains. The Δ*ureG* strain was complemented with the native *ureG* gene by ectopic integration.

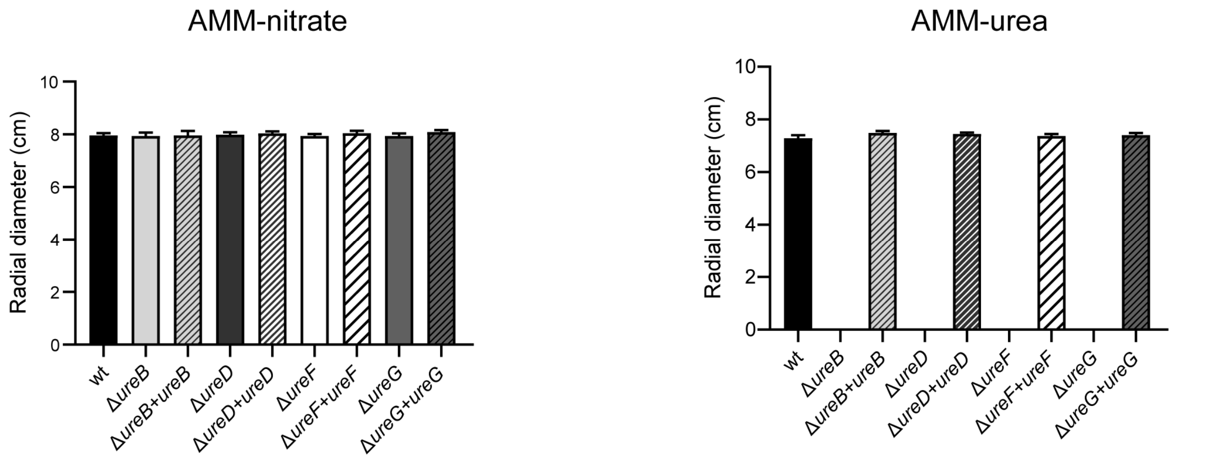

Fig S12. Radial diameter of *A. fumigatus* wild type, Δ*ureB,* Δ*ureD,* Δ*ureF and* Δ*ureG* mutants and their corresponding complemented strains grown on AMM nitrate or AMM urea from 5×10^3 spores for 7 days at 37°C. Results are expressed as the average radial diameters of three repetitions ± standard deviations

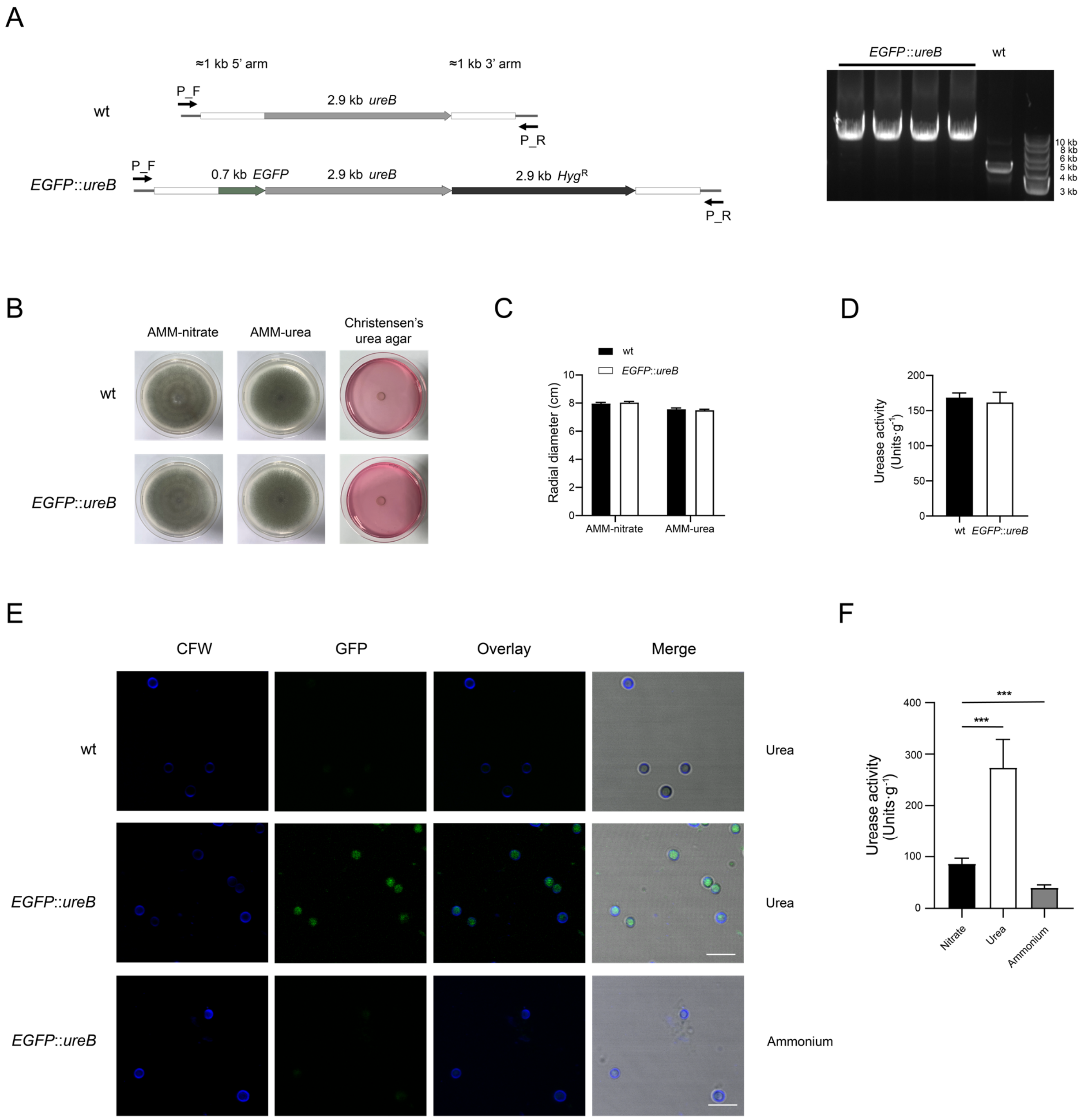

Fig S13. Construction and phenotypic examination of *EGFP*::*ureB* strain. (A) Genome organization and diagnostic PCR analysis of *EGFP*::*ureB*. (B) Growth and urease activity tests on AMM and Christensen's urea agar. (C) Radical diameter measured after 7 days of inoculation with 5×10^3 spores on AMM nitrate/urea agar. (D) Urease activity of cell-free extracts from wild type and *EGFP*::*ureB*. (E) The fluorescent signal of EGFP-UreB was observed by confocal microscopy in *EGFP*::*ureB* conidia harvested on AMM urea and AMM ammonium. Scale bar, 10 µm. (F) Conidial urease activity of *EGFP*::*ureB* harvested under different nitrogen sources. Statistical significance was assessed using a one-way ANOVA test with multiple comparisons; ***$P < 0.001$.

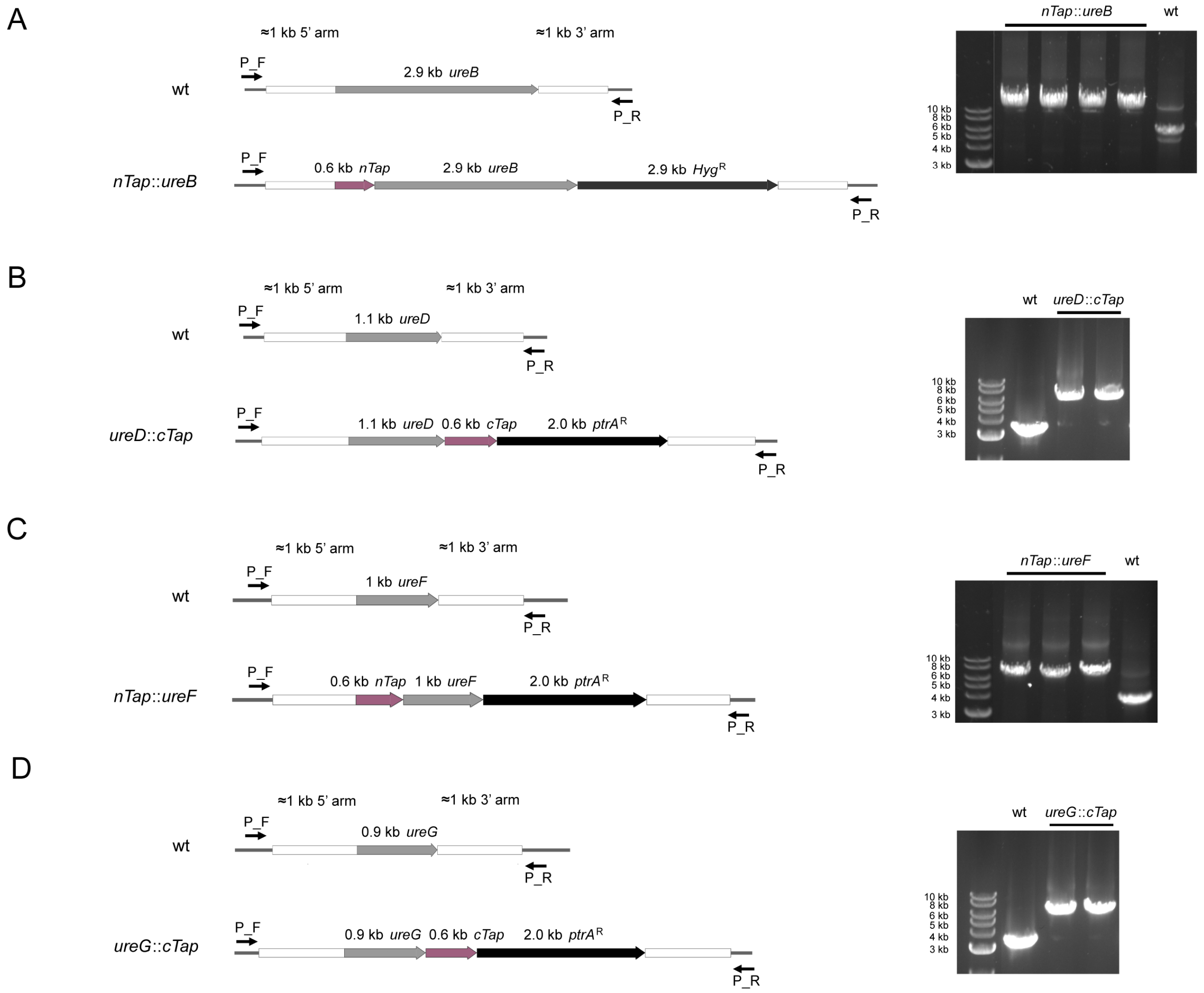

Fig S14. Construction of Tap-tagged strains. Genome organization and diagnostic PCR analyses of Tap-tagged strains of *ureB* (A), *ureD* (B), *ureG* (C), *ureF* (D).

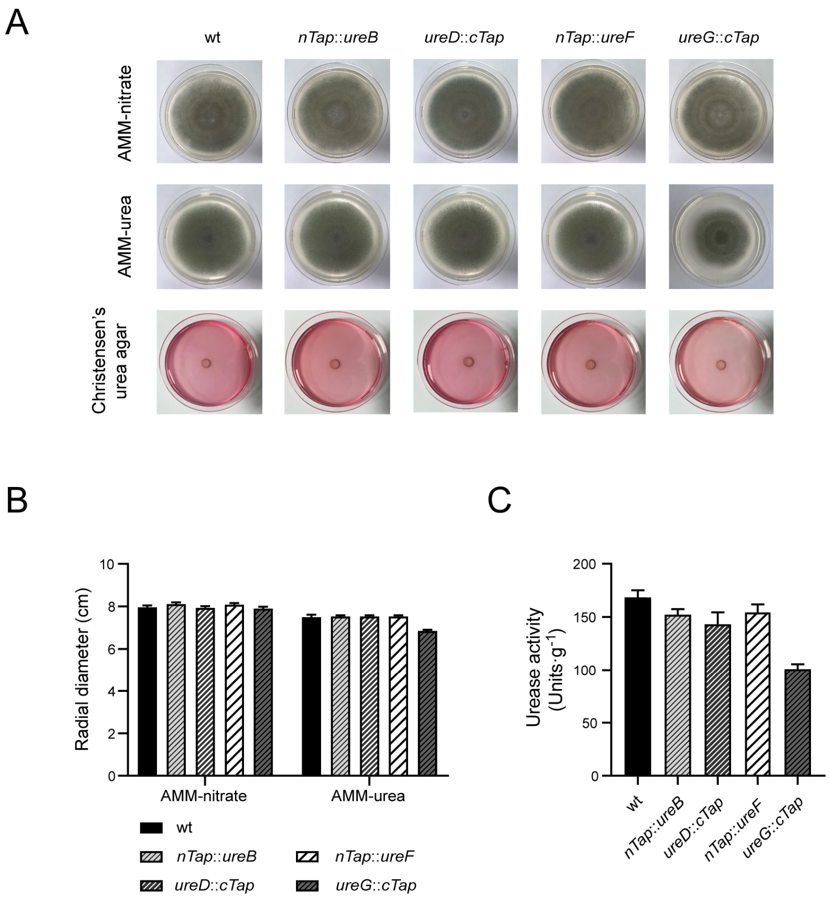

Fig S15. Phenotypic examination of Tap-tagged strains of *ure*B, *ure*D, *ure*G, *ureF*. (A) Growth and urease activity tests on AMM and Christensen's urea agar. (B) Radical diameter measured after 7 days of inoculation with 5×10^3 spores on AMM nitrate/urea agar. (C) Urease activity of cell-free extracts from wild type and the Tap-tagged strains.

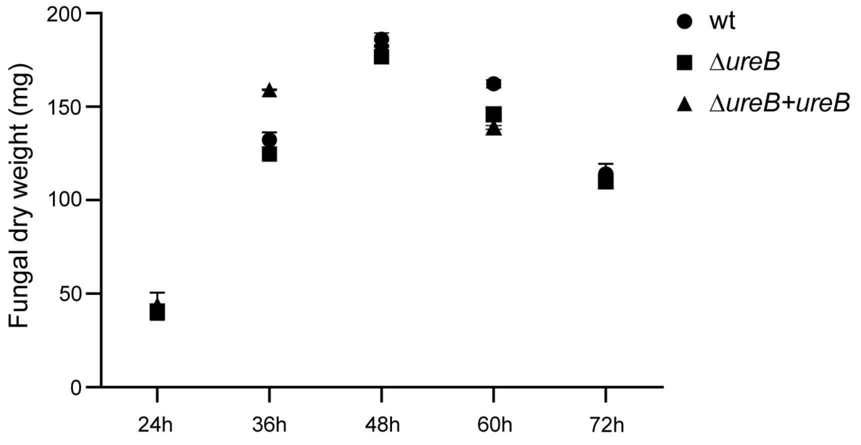

Fig S16. Growth curve of wild-type, Δ*ureB* mutant and complemented strains in AMM nitrate.

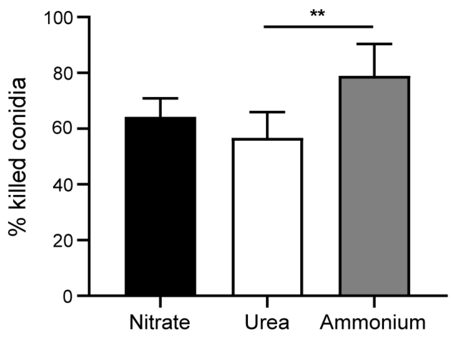

Fig S17. Susceptibility of *A. fumigatus* conidia harvested under different nitrogen sources to macrophage-mediated killing.

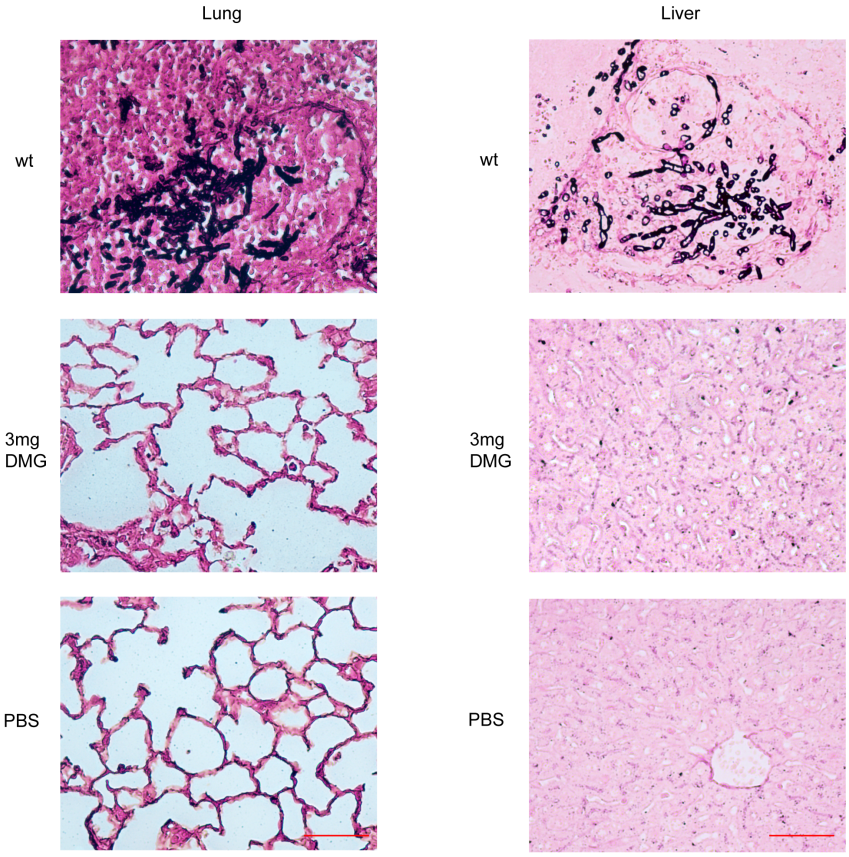

Fig S18. Histopathology of lungs and livers from the intratracheal/intravenous infection models treated with DMG. Representative lung and liver sections stained with GMS (Grocott's Methenamine Silver) exhibit fungal colonization (shown as black hyphae). Scale bar, 50 μm.

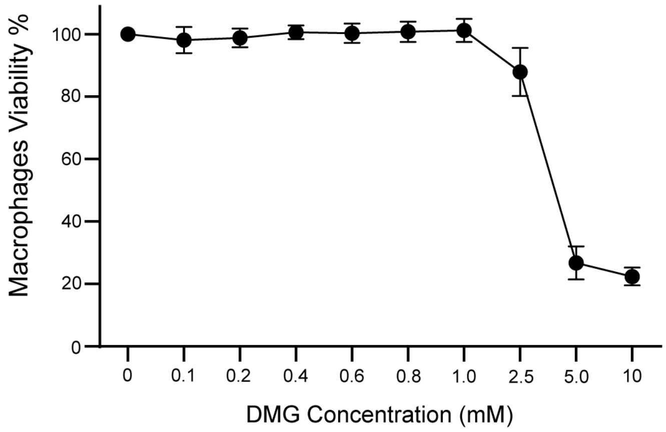

Fig S19. Effect of different concentrations of dimethylglyoxime (DMG) on macrophage viability. Macrophage viability was not affected by DMG with a concentration ≤1 mM, but decreased when DMG concentration was > 1 mM.

References

1. Xu J, Gong ZZ. 2003. Intron requirement for AFP gene expression in Trichoderma viride. Microbiology 149:3093-3097.
2. Krappmann S, Jung N, Medic B, Busch S, Prade RA, Braus GH. 2006. The *Aspergillus nidulans* F-box protein GrrA links SCF activity to meiosis. Mol Microbiol 61:76-88.
3. Busch S, Schwier EU, Nahlik K, Bayram Ö, Helmstaedt K, Draht OW, Krappmann S, Valerius O, Lipscomb WN, Braus GH. 2007. An eight-subunit COP9 signalosome with an intact JAMM motif is required for fungal fruit body formation. Proc Natl Acad Sci U S A 104:8089.
4. Busso D, Peleg Y, Heidebrecht T, Romier C, Jacobovitch Y, Dantes A, Salim L, Troesch E, Schuetz A, Heinemann U, Folkers GE, Geerlof A, Wilmanns M, Polewacz A, Quedenau C, Büssow K, Adamson R, Blagova E, Walton J, Cartwright JL, Bird LE, Owens RJ, Berrow NS, Wilson KS, Sussman JL, Perrakis A, Celie PHN. 2011. Expression of protein complexes using multiple *Escherichia coli* protein co-expression systems: a benchmarking study. J Struct Biol 175:159-170.
5. Le SQ, Gascuel O. 2008. An improved general amino acid replacement matrix. Mol Biol Evol 25:1307-1320.
6. Tamura K, Stecher G, Kumar S. 2021. MEGA11: Molecular Evolutionary Genetics Analysis Version 11. Mol Biol Evol 38:3022-3027.
